# Genetic diversity and relationship of cattle populations of East India: distinguishing lesser known cattle populations and established breeds based on STR markers

**DOI:** 10.1186/2193-1801-2-359

**Published:** 2013-07-30

**Authors:** Rekha Sharma, Avishek Maitra, Pramod Kumar Singh, Madhu Sudan Tantia

**Affiliations:** National Bureau of Animal Genetic Resources, Karnal, 132 001 Haryana India

**Keywords:** Conservation, Diversity, Genetic relationship, Indian Cattle, Microsatellite markers, Population structure

## Abstract

India has 34 recognized breeds of cattle in addition to many more not characterized and accredited so far. It is imperative to characterize all the cattle germplasm of the country so as to have better breeding and conservation options. Thus, present study was planned for assessing genetic diversity and relationship between three local cattle populations (Gangatiri, Shahabadi and Purnea) and two established cattle breeds (Bachaur and Siri) of eastern India by using 21 FAO and ISAG recommended microsatellite markers. A total of 243 unrelated DNA samples of five cattle populations were collected from respective habitats. A total of 304 microsatellite alleles were identified with number of alleles at one locus ranging from 5 to 29. The average observed heterozygosity lie within the narrow range of 0.681 ± 0.04 in Purnea to 0.721 ± 0.03 in Siri. Mean estimates of observed and expected heterozygosity over all loci and breeds were 0.704 ± 0.02 and 0.720 ± 0.01, respectively. In the overall population, the homozygote excess (F_IT_) of 0.073 ± 0.02, was partly due to the homozygote excess within breeds (F_IS_ = 0.026 ± 0.02) and to a larger extent due to genetic differentiation among breeds (F_ST_ = 0.048 ± 0.01). The genetic distance, STRUCTURE and Principal Component Analyses concluded that the Siri cattle are most distinct among the investigated cattle populations. Furthermore the analysis of genetic structure indicated that the most probable number of clusters is four. All analysis showed that a significant amount of genetic variation is maintained in local cattle populations of which Shahabadi and Purnea are distinct from the recognized breeds of the area and needs recognition as breeds.

## Introduction

A large and divergent range of agro-ecological zones in India has helped to develop number of cattle populations. Cattle are the largest livestock species in India and constitute 37.5% of its total livestock population (BAHS-Basic Animal Husbandry Statistics 2012). However, it is surprising to note that only 11.6% of total cattle (199.08 million) belong to pure indigenous breeds, whereas, 69.7% were classified as non-descript indigenous animals (BAHS-Basic Animal Husbandry Statistics 2012). Non-descript population includes the cross-bred populations, populations which are mixture of different breeds or the populations which have not yet been studied or described. Non-descript population is greatly contributing to the total Gross Domestic Product from livestock sector. In addition, indigenous animal genetic resources is known for heat and draught tolerance, disease resistance and subsistence on poor feed, opening scope for allele mining for these traits. Thus the emphasis should be to describe, characterize and document lesser known populations in the country so that the proportion of non-descript population is considerably transformed in to defined breeds. This will be first step towards planning the organized breeding program for their genetic improvement, conservation strategies and sustainable utilization.

**Figure 1:**
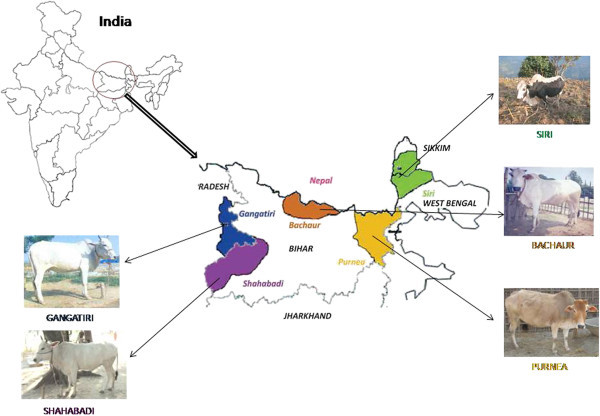
**Distribution of cattle populations.**

Gangatiri, Shahabadi and Purnea are three non-descript cattle populations of eastern India. The registered cattle breeds in and surrounding area include Bachaur and Siri. Bachaur, Shahabadi and Purnea cattle are from Bihar state whereas, Gangatiri belongs to area of Uttar Pradesh state bordering Bihar and habitat of Siri borders Bihar state on eastern side (Figure 1). Typical animals of these breeds and populations are available in their native tracts in optimum number (>4000) to constitute viable breeding populations (FAO 2007). Since last two decades these populations are showing declining trend (Singh 2009) thus need immediate attention, failing which our ancestral efforts of artificial selection, operative since time immemorial, may go in vein and there may be a non-repairable loss from the national and world gene pool. Employment of microsatellite markers is one of the most powerful means for studying the genetic diversity, calculation of genetic distances, detection of bottlenecks and admixture because of their high degree of polymorphism, random distribution across the genome and neutrality with respect to selection (Dodgson *et al.*^1997^). Considering the importance of cattle in Indian agriculture, efforts have been made to evaluate the genetic diversity and relationship in Indian cattle using microsatellite markers. These included native cattle breeds adapted to the north-western arid and semi-arid region of India (Sodhi *et al.*^2008^; ^2011^), north Indian cattle breeds (Sharma *et al.*^2009^), South Indian breeds (Metta *et al.*^2004^) and cattle of Orissa state and hill cattle of Kumaun (Sharma *et al.*2012a). However, the genetic relationship between native cattle breeds of eastern India is unknown.

The present study aimed to genetically characterize and distinguish lesser known cattle populations (Shahabadi, Purnea and Gangatiri) and recognized cattle breeds of eastern India (Bachaur and Siri). Twenty one microsatellites (Short Tandem Repeats, STR) were amplified in five multiplex PCR. We intend to evaluate not only the current diversity but also to know their relationship for the conservation of the genetic diversity in the context of biodiversity management programs.

## Materials and methods

### Biological material

Shahabadi, Purnea and Gangatiri are non-descript cattle from Eastern India (Figure 1). Gangatiri and Shahabadi are white or grey colored animals. Gangatiri animals are reared for milk production (4–6 liters per day) as well as for agricultural operations (Singh *et al.*^2007^). Shahabadi cattle are mainly distributed in Buxar, Bhojpur, Kaimoor and Rohtas (Sasaram) districts of Bihar. The milk production varies from 2 to 6 liters in a day (Sharma *et al.*2012b). Purnea animals are of small size and compact body. The coat colour of Purnea cattle varies from light to deep red with few greyish white animals (Sharma *et al.*^2013^). The animals are primarily employed for agricultural operations, for carrying load and transportation. The habitat of Purnea cattle encompasses Purnia commissionery of Bihar state (Purnia, Araria, Katihar and Kishanganj districts).

Bachaur and Siri are recognized cattle breeds of Eastern India. Bachaur, a famous draught purpose cattle breed is concentrated in Bachaur Pargana (Madhubani, Sitamarhi and Darbhanga districts) of Bihar state. The habitat of Bachaur has shrunken with time to northern part of Bihar bordering Nepal. The animals are white to grey in colour. The cows are poor milker, producing an average of 2.2 kg of milk per day and are managed under extensive management (Sharma *et al.*^2007^). Animals of Siri breed are small in size and are distributed in the hilly tracts of West Bengal (adjoining state of Bihar) and Sikkim states of India. The color most frequently seen is black and white or extensive solid black. The Siri has a hump that is thoracic, muscular-fatty and slightly forward in position as compared to other Zebu breeds of India. The animal carries a thick coat all the year around which protects them from heavy rains and severe cold (Sharma *et al.*^2008^).

### Blood sampling and microsatellite analysis

Samples of the populations included in this study represented animals of the original autochthonous phenotype. Blood samples from 243 individuals were collected (Bachaur-50, Gangatiri-50, Shahabadi-48, Purnea-47 and Siri-48) from different villages of habitat (Figure 1) while avoiding closely related individuals on the basis of detailed interview with owners. Blood samples collected in 10 ml vacuitainer tubes containing EDTA as anticoagulant were stored at −20°C until DNA extraction. DNA isolation was carried out using modified Phenol-chloroform method (Sambrook *et al.*^1989^). Isolated DNA samples were amplified by PCR in correspondence with the selected panel of 21 loci. The loci were chosen, according to ISAG/FAO recommendation aiming to analyze high polymorphic markers spread all over the genome and ability to co-amplify in PCR reactions (FAO [Food and Agricultural Organization of the United Nations] 2011). The fluorochrome labeled (FAM, NED, PET& VIC) primers were synthesized by Applied Biosystems (Table 1). For amplification, 50-100 ng of genomic DNA was added to a reaction mixture containing 50 pMol of primer- forward and reverse, 200 μM of every dNTPs, 1.5 mM of MgCl_2_ and 0.5U of *taq* polymerase in a final volume of 25 μl. All the microsatellites were amplified by a MJ thermal cycler at the following conditions: initial denaturation step of 1 min at 95°C, 30 cycles of 1 min at 95°C, 1 min at T°C (optimum annealing temperature of each primer) and 1 min at 72°C and a final extension of 5 min at 72°C. Amplified fragments were separated by capillary electrophoresis using an ABI PRISM 310 automatic sequencer (Applied Biosystems, Foster City, CA, USA). Fluorescently labeled fragments were detected and sized using GeneMapper (version 3.7, Applied Biosystems).

**Table 1:** **Characteristics of 21 microsatellite loci used in present study**

Primers	Primer sequences (5′-3′)	Forward label	Set	Annealing temp	Product size (bp)	Total number of alleles per locus
BM1824	gagcaaggtgtttttccaatc	VIC	4	58°C	178-194	7
	cattctccaactgcttccttg					
CSSM08	cttggtgttactagccctggg	VIC	3	55°C	182-200	7
	gatatatttgccagagattctgca					
CSSM33	cactgtgaatgcatgtgtgtgagc	NED	5	58°C	148-186	19
	cccatgataagagtgcagatgact					
CSSM66	acacaaatcctttctgccagctga	FAM	4	60°C	167-207	17
	aatttaatgcactgaggagcttgg					
ETH10	gttcaggactggccctgctaaca	NED	1	58°C	185-221	14
	cctccagcccactttctcttctc					
ETH225	gaacctgcctctcctgcattgg	VIC	4	64°C	134-156	11
	actctgcctgtggccaagtagg					
ETH3	gatcaccttgccactatttcct	NED	4	57°C	92-122	14
	acatgacagccagctgctact					
HEL09	cccattcagtcttcagaggt	FAM	5	59°C	140-168	13
	cacatccatgttctcaccac					
HEL5	gcaggatcacttgttaggga	VIC	3	55°C	137-187	20
	agacgttagtgtacattaac					
ILSTS06	tgtctgtatttctgctgtgg	FAM	5	58°C	279-303	11
	acacggaagcgatctaaacg					
ILSTS11	gcttgctacatggaaagtgc	NED	1	58°C	261-269	5
	ctaaaatgcagagccctacc					
ILSTS34	aagggtctaagtccactggc	VIC	5	59°C	138-208	29
	gacctggtttagcagagagc					
ILSTS33	tattagagtggctcagtgcc	PET	3	55°C	137-163	10
	atgcagacagttttagaggg					
INRA05	caatctgcatgaagtataaatat	FAM	2	54°C	130-144	8
	cttcaggcataccctacacc					
INRA35	atcctttgcagcctccacattg	FAM	3	54°C	80-142	24
	ttgtgctttatgacactatccg					
INRA63	atttgcacaagctaaatctaacc	PET	2	54°C	164-188	11
	aaaccacagaaatgcttggaag					
MM12	caagacaggtgtttcaatct	PET	4	52°C	88-132	20
	atcgactctggggatgatgt					
MM8	cccaaggacagaaaagact	NED	2	55°C	114-140	10
	ctcaagataagaccacacc					
TGLA122	ccctcctccaggtaaatcagc	VIC	1	58°C	135-179	18
	aatcacatggcaaataagtacatac					
TGLA227	cgaattccaaatctgttaatttgct	PET	2	55°C	97-119	16
	acagacagaaactcaatgaaagca					
TGLA53	gctttcagaaatagtttgcattca	FAM	1	58°C	142-184	20
	atcttcacatgatattacagcaga					

### Statistical analysis

GENALEX 6.2 software (Peakall and Smouse, 2008) was used to estimate basic population genetic descriptive statistics for each marker and population: gene frequency, observed number of alleles (N_o_), number of private alleles, effective number of alleles (N_e_), observed (H_o_) and expected heterozygosity (H_e_) and Hardy-Weinberg equilibrium (HWE). Wright’s statistics F_IS_ (f), F_ST_ (θ) and F_IT_ (F) and Nei’s (Nei 1987) standard genetic distances among populations were calculated as implemented in GENALEX software. Pair wise matrix of the genetic distances was then used to obtain a Neighbor-joining tree which was visualized using the software TreeView (Page 1996). Bootstraps of 1000 replicates were performed in order to test the robustness of tree topology using the Phylip software (Felsenstein 1993). Multivariate analysis of microsatellite allele frequencies (Principal Component analysis, PCA) was applied to reveal the underlying evolutionary history and admixture among populations. An alternative model-based Bayesian clustering analysis was used to infer how many clusters or subpopulations (K) were most appropriate for interpreting the data without prior information on the number of locations at which the individuals were sampled as implemented in STRUCTURE v2.2 (Pritchard *et al.*^2000^). Analysis was performed with a burn-in length of 50,000 followed by 30,000 MCMC (Marcov Chain Monte Carlo) iterations for each K = 1 to 7 with five replicate runs for each K using independent allele frequencies and no admixture. Optimal K value was selected after analyzing the result files with STRUCTURE Harvester (Earl and vonHoldt 2012).

## Results and discussion

In the present study genetic status and diversity of lesser known indigenous cattle populations of eastern India and their relationship with established breeds of the same region was established using microsatellite markers. All microsatellite markers used in this study were successfully amplified in five multiplex sets designed with consideration for annealing temperature, product size and specific dye label in all the populations (Table 1). The genotype data generated in present study showed that significant amount of genetic variation is maintained in local cattle populations. All the markers were found to be polymorphic in each of the five populations analyzed. Considering all the populations, majority of the markers were in HWE. Number of loci deviating from HWE (P < 0.05) were one for Gangatiri (ETH3) and Shahabadi (MM8), two for Bachaur (TGLA122, MM12), three for Siri (TGLA227, ETH10, CSSM66) and none for Purnea cattle populations. The level of variations depicted by number of alleles at each locus serves as a measure of genetic variability having direct effect on differentiation of breeds within a species (Buchanan et al. 1994). All the 21 microsatellite loci showed ample polymorphism for evaluating within breed genetic variability and exploring genetic differences between breeds. A total of 304 alleles were detected with ILSTS34 presenting the highest number of alleles per locus (29) while ILSTS11 presented the lowest (5) number of alleles (Table 1). ILSTS34 presented the highest number of alleles per locus (19) in Bachaur cattle while BM1824, ETH10 and ILSTS11 presented the lowest (4) in Purnea cattle. The effective number of alleles per locus in a population varied from 1.492 (TGLA227) to 10.301 (INRA35) and was proportional to the value of expected heterozygosity found in these loci (TGLA227, 0.330 and INRA35, 0.903) (Table 2). Lower values of expected number of alleles as compared to observed number of alleles in all the populations suggested that there were many low frequency alleles in the populations. The mean observed number of alleles across all the loci was 9.486 ± 0.327 and was higher than other indigenous cattle breeds (Metta *et al.*^2004^; Mukesh *et al.*^2004^; Pandey *et al.*2006a and 2006b). Lower allelic diversity than studied populations have also been reported in exotic cattle- Burlina-6.7 (Dalvit *et al.*^2008^), Brown Swiss-5.4 (Schmid *et al.*^1999^) and Creole cattle-7.2 (Egito *et al.*^2007^). Previously also the allelic diversity in the Indian livestock breeds has been observed to be higher than that reported for the European counterpart (Joshi *et al.*^2012^) that has been attributed to lack of artificial selection pressure. Allelic diversity of similar magnitude has also been reported in Tharparkar, Rathi and Orissa cattle populations of India (Sodhi *et al.*^2008^; Sharma *et al.*2012a). Measures of genetic diversity based on allelic richness are considered important in conservation genetics as marker-assisted methods for maximizing number of alleles conserved have been shown to be effective (Bataillon *et al.*^1996^). It is also relevant in long-term perspective, as selection limits are determined by the initial allelic composition rather than by heterozygosity (Petit et al. 1998).

**Table 2:** **Genetic variability parameters of five cattle populations**

	Bachaur				Gangatiri				Shahabadi				Purnea				Siri			
Locus	No	Ne	Ho	He	No	Ne	Ho	He	No	Ne	Ho	He	No	Ne	Ho	He	No	Ne	Ho	He
**BM1824**	6	2.678	0.580	0.627	5	2.400	0.660	0.583	6	2.471	0.532	0.595	4	2.297	0.404	0.565	6	3.683	0.667	0.729
**CSSM08**	7	2.119	0.510	0.528	6	2.533	0.667	0.605	6	2.496	0.545	0.599	7	2.409	0.581	0.585	6	2.950	0.600	0.661
**CSSM33**	14	6.767	0.875	0.852	15	6.840	0.860	0.854	16	7.810	0.896	0.872	13	7.007	0.826	0.857	12	5.914	0.830	0.831
**CSSM66**	9	3.740	0.680	0.733	11	4.803	0.860	0.792	13	5.086	0.854	0.803	14	5.084	0.745	0.803	8	3.411	0.688	0.707
**ETH10**	9	4.655	0.880	0.785	11	5.025	0.900	0.801	6	4.064	0.814	0.754	4	2.673	0.891	0.626	14	7.266	0.894	0.862
**ETH225**	9	2.231	0.560	0.552	8	2.019	0.500	0.505	10	2.051	0.583	0.512	10	6.258	0.851	0.840	9	4.148	0.708	0.759
**ETH3**	8	3.541	0.980	0.718	9	3.671	0.860	0.728	14	6.508	0.958	0.846	7	3.899	0.979	0.744	8	3.796	0.979	0.737
**HEL09**	10	7.529	0.979	0.867	10	6.527	0.900	0.847	10	7.396	0.896	0.865	10	8.000	0.833	0.875	12	8.243	0.766	0.879
**HEL5**	11	4.361	0.714	0.771	10	5.219	0.857	0.808	13	6.585	1.000	0.848	10	3.901	0.844	0.744	10	6.853	0.756	0.854
**ILSTS06**	7	3.058	0.500	0.673	8	4.062	0.640	0.754	9	3.298	0.545	0.697	8	4.691	0.619	0.787	9	4.485	0.489	0.777
**ILSTS11**	5	2.288	0.469	0.563	5	2.600	0.500	0.615	5	3.150	0.558	0.683	4	1.928	0.422	0.481	5	4.212	0.638	0.763
**ILSTS34**	19	7.444	0.875	0.866	16	6.046	0.760	0.835	15	4.571	0.750	0.781	10	4.054	0.652	0.753	14	6.914	0.851	0.855
**ILSTS33**	8	3.460	0.714	0.711	7	2.350	0.548	0.575	9	4.150	0.727	0.759	6	2.864	0.531	0.651	8	4.295	0.733	0.767
**INRA05**	7	4.367	0.640	0.771	8	4.394	0.820	0.772	7	4.509	0.708	0.778	6	4.251	0.581	0.765	7	3.938	0.729	0.746
**INRA35**	12	5.723	0.755	0.825	8	4.570	0.667	0.781	17	10.301	0.860	0.903	6	3.081	0.621	0.675	8	4.054	0.844	0.753
**INRA63**	6	2.501	0.520	0.600	5	2.627	0.580	0.619	6	2.781	0.563	0.640	7	3.446	0.837	0.710	7	2.366	0.521	0.577
**MM12**	11	5.149	0.760	0.806	10	4.146	0.720	0.759	13	4.295	0.729	0.767	15	4.909	0.787	0.796	10	2.841	0.604	0.648
**MM8**	8	2.737	0.560	0.635	6	2.667	0.680	0.625	8	3.072	0.625	0.674	8	2.703	0.605	0.630	6	2.525	0.583	0.604
**TGLA122**	14	8.666	0.900	0.885	12	9.728	0.920	0.897	14	9.732	0.791	0.897	13	7.627	0.844	0.869	12	8.925	0.915	0.888
**TGLA227**	6	1.492	0.380	0.330	8	1.571	0.360	0.363	12	1.831	0.417	0.454	10	1.871	0.302	0.466	11	2.869	0.667	0.651
**TGLA53**	13	3.408	0.740	0.707	15	2.648	0.640	0.622	14	3.485	0.628	0.713	15	2.557	0.545	0.609	12	3.532	0.681	0.717
**Mean**	**9.476**	**4.186**	**0.694**	**0.705**	**9.190**	**4.117**	**0.709**	**0.702**	**10.619**	**4.745**	**0.713**	**0.735**	**8.905**	**4.072**	**0.681**	**0.706**	**9.238**	**4.629**	**0.721**	**0.751**
**S. D.**	**0.752**	**0.440**	**0.038**	**0.030**	**0.716**	**0.436**	**0.034**	**0.030**	**0.824**	**0.532**	**0.035**	**0.027**	**0.771**	**0.402**	**0.040**	**0.027**	**0.589**	**0.421**	**0.028**	**0.020**

Estimates of observed heterozygosity including all loci and population (0.704 ± 0.016) confirmed the remarkable level of diversity in the studied populations. Among populations, observed heterozygosity ranged from 0.681 ± 0.04 to 0.721 ± 0.028 with the lowest value found in Purnea cattle and the highest in Siri cattle. Most of the indigenous breeds including Kherigarh-0.57 (Pandey *et al.*2006a), Kenkatha-0.54 (Pandey *et al.*2006b), Sahiwal-0.43 (Mukesh *et al.*^2004^) and Deoni-0.59 (Metta *et al.*^2004^) showed lower estimates of observed heterozygosity than local cattle populations and the breeds investigated in present study. Overall heterozygosity estimates were comparable with Tharparkar cattle (0.64, Sodhi *et al.*^2008^), Orissa cattle populations (0.62 to 0.66, Sharma *et al.*2012a) of India, Chinese cattle (0.62, Sun *et al.*^2008^) and Creole cattle (0.61, Egito *et al.*^2007^). Purnea cattle represented the lowest observed and effective number of alleles as well as observed and expected heterozygosity. On the other hand Siri cattle presented the highest value of the above said parameters amongst all genetic groups (Table 2). Genetic variation is necessary to allow organisms to adapt to ever changing environments with some of this variation stemming from introduction of new alleles by the random and natural process of mutation. Higher genetic variation in Siri cattle must have contributed to its adaptability and this is reflected in wide-spread distribution of Siri cattle not only in India but also in neighboring country, Bhutan.

Observed heterozygosity was lower than expected heterozygosity in Bachaur, Shahabadi, Purnea and Siri cattle populations, showing departure from HWE and analysis of F_IS_ evidenced some heterozygote deficiency too (Table 3). This disequilibrium was caused by heterozygote deficiency in each population which was highest in Purnea (0.042 ± 0.041) and lowest in Bachaur (0.017 ± 0.030). On the contrary, Gangatiri cattle presented slight heterozygote excess in the population (−0.010 ± 0.022) which was expressed in heterozygosity pattern too (H_o_ = 0.709 ± 0.034, H_e_ = 0.702 ± 0.030) (Table 2). Existence of this population in small geographical region with free grazing of Gangatiri and non-descript animals in a herd could be the likely sources for the sufficient heterozygotes. Positive F_IS_ estimate for Bachaur, Shahabadi, Purnea and Siri indicated either the presence of inbreeding and /or Wahlund effect (presence of population substructure within breed). Since blood samples were collected from different villages, presence of a hidden substructure cannot be ruled out. In fact, animals of the same population but belonging to different villages could derive from genetically different founders. A significant homozygote excess was also observed in other studies on indigenous cattle breeds such as Sahiwal F_IS_ = 0.32 (Mukesh *et al.*^2004^). Most likely inbreeding in these populations is arising from unplanned and unsystematic breeding owing to lack of sufficient number of breeding males required in the breeding region. Moreover instead of local bull semen, exotic (Jersey and Holstein Friesian) or crossbred semen was available in the habitat. This can be one of the causes for dilution of the populations. Together these two factors were resulting in the reduction of true to the breed type animals. Actual picture of Bachaur cattle in its habitat also indicated towards the inbreeding in the population. Very less number of breeding bulls in the habitat of Bachaur cattle has been reported (Singh 2004). Bachaur is a draft purpose breed thus most of the males are used in carrying loads and agricultural operations. These males are castrated at the age of one year leading to their genetic death. Similarly over the last few years the population of Siri cattle has been declining due to extensive cross breeding with the result that Siri animals are now confined only to the remote and inaccessible areas of Sikkim. Thus, few Siri breeding bulls have been left in the habitat which might decrease the effective population size.

**Table 3:** **Heterozygote deficiency (F) of five cattle populations of India**

Locus	Bachaur	Gangatiri	Shahabadi	Purnea	Siri
**BM1824**	0.074	−0.131	0.106	0.284	0.085
**CSSM08**	0.034	−0.102	0.090	0.007	0.092
**CSSM33**	−0.027	−0.007	−0.027	0.036	0.001
**CSSM66**	0.072	−0.086	−0.063	0.073	0.027
**ETH10**	−0.121	−0.124	−0.080	−0.424	−0.036
**ETH225**	−0.015	0.010	−0.139	−0.013	0.067
**ETH3**	−0.366	−0.182	−0.132	−0.316	−0.329
**HEL09**	−0.129	−0.063	−0.036	0.048	0.128
**HEL5**	0.073	−0.060	−0.179	−0.135	0.115
**ILSTS06**	0.257	0.151	0.217	0.213	0.370
**ILSTS11**	0.166	0.188	0.182	0.123	0.163
**ILSTS34**	−0.011	0.089	0.040	0.134	0.005
**ILSTS33**	−0.005	0.047	0.042	0.184	0.044
**INRA05**	0.170	−0.062	0.090	0.240	0.023
**INRA35**	0.085	0.147	0.047	0.081	−0.121
**INRA63**	0.134	0.064	0.122	−0.179	0.098
**MM12**	0.057	0.051	0.050	0.011	0.068
**MM8**	0.118	−0.088	0.073	0.040	0.034
**TGLA122**	−0.017	−0.025	0.119	0.028	−0.030
**TGLA227**	−0.153	0.009	0.082	0.351	−0.023
**TGLA53**	−0.047	−0.028	0.119	0.104	0.050
**Mean**	**0.017**	**−0.010**	**0.034**	**0.042**	**0.040**
**S. D.**	**0.030**	**0.022**	**0.023**	**0.041**	**0.028**

Results of F-statistics for each of the 21 loci across populations are presented in Table 4. The global deficit of heterozygotes across populations (F_IT_) amounted to 7.3% (P < 0.001). An overall significant deficit of heterozygotes (F_IS_) of 2.6% occurred in the analyzed loci because of inbreeding within populations. All loci except six contributed to heterozygote deficit within populations. The studied populations showed a low but significant genetic differentiation among five populations (F_ST_ = 0.048). All loci contributed to the differentiation with the highest values found for ETH25 (19.6%). Genetic differentiation of similar magnitude has been reported among cattle breeds of Orissa and hill cattle of Kumaun (0.044) from India (Sharma *et al.*2012a). Much higher F_ST_ value have been reported in other indigenous cattle (Sodhi *et al.*^2011^). While, several reports on exotic cattle viz. North European breeds F_ST_ =0.107 (McHugh *et al.*^1998^), seven European cattle breeds F_ST_ =0.112 (Kantanen *et al.*^2000^) and Swiss cattle F_ST_ = 0.090 (Schmid *et al.*^1999^) also depicted higher genetic differentiation than populations investigated in this study. The low value of genetic differentiation in Eastern Indian cattle may be attributed to the lack of high selection pressure as compared to established breeds of India or cattle breeds of developed countries. These results reflect that within-breed genetic variations is more than between-breed and this variation could be a valuable tool for genetic improvement and conservation of cattle populations of eastern India.

**Table 4:** **Global F-statistics and estimates of Nm across five cattle populations**

Locus	Fis	Fit	Fst	Nm
**BM1824**	0.083	0.126	0.047	5.025
**CSSM08**	0.025	0.094	0.070	3.303
**CSSM33**	−0.005	0.033	0.037	6.438
**CSSM66**	0.003	0.014	0.011	21.733
**ETH10**	−0.144	−0.088	0.049	4.834
**ETH225**	−0.011	0.188	0.196	1.023
**ETH3**	−0.261	−0.208	0.042	5.726
**HEL09**	−0.010	0.017	0.027	9.078
**HEL5**	−0.036	0.053	0.086	2.648
**ILSTS06**	0.242	0.256	0.017	14.078
**ILSTS11**	0.166	0.229	0.075	3.091
**ILSTS34**	0.049	0.097	0.051	4.697
**ILSTS33**	0.060	0.119	0.063	3.732
**INRA05**	0.092	0.098	0.007	36.707
**INRA35**	0.048	0.159	0.116	1.905
**INRA63**	0.040	0.056	0.017	14.799
**MM12**	0.046	0.065	0.019	12.821
**MM8**	0.036	0.048	0.012	21.263
**TGLA122**	0.015	0.033	0.018	13.656
**TGLA227**	0.061	0.084	0.025	9.857
**TGLA53**	0.040	0.053	0.013	18.427
**Mean**	0.026	0.073	0.048	10.230
**SE**	0.022	0.022	0.010	1.922

**Figure 2:**
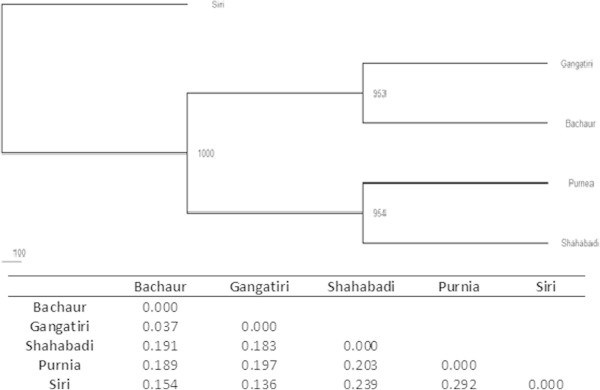
**Genetic relationship among cattle populations using Nei’s distance.** The number in the branch indicates the percentage occurrence in 1000 bootstrap replicates. Table shows Nei’s genetic distance between populations and breeds analyzed.

**Figure 3:**
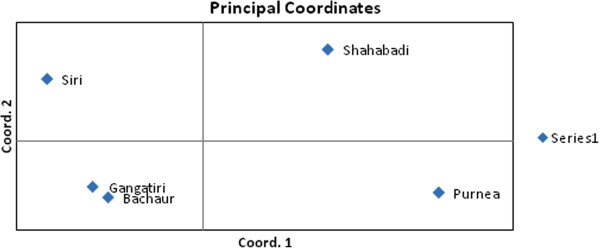
**Principal Coordinates Analysis (PCA) via Covariance matrix with data standardization.**

Pair-wise genetic differentiations quantified by F_ST_ estimates identified Bachaur and Gangatiri (0.008) as the closest populations while the most differentiated were Purnea and Siri (0.044) (Table 5). Similar results were obtained with Nei’s genetic distance matrix. The highest genetic distance was found between Purnea and Siri (0.292), while Bachaur and Gangatiri were closest to each other (0.037) (Figure 2). In accordance to these observations highest gene flow was among the Bachaur and Gangatiri (N_m_ = 32.809) populations and least between Purnea and Siri (N_m_ = 5.408). Overall N_m_ value also indicated high rate of genetic flow between the populations (10.230 ± 1.922) (Table 4). Visualization of breed relationship was done by constructing Neighbor joining tree on the basis of Nei’s genetic distance. As expected, the Siri was most distinct and separated first, while remaining populations formed two groups with clustering of Gangatiri and Bachaur on one node and Purnea and Shahabadi on second with more than 95% bootstrap value (Figure 2). This grouping pattern was further supported by PCA analysis. Since phylogenetic reconstruction may not take into account the effects of admixture between breeds, PCA was performed to further investigate possible genetic relationship between recognized breeds and local populations. First three dimension of the PCA (PC1 = 40.35; PC2 = 30.72; PC3 = 22.74) accounted for 93.81% of total variation. In the multivariate space defined by PCA, Bachaur and Gangatiri were much closer. It confirmed distinctiveness of Siri as well as lesser known cattle populations of Purnea, and Shahabadi (Figure 3).

**Table 5:** **Pair wise population matrix of Fst values between analyzed populations**

	Bachaur	Gangatiri	Shahabadi	Purnia	Siri
**Bachaur**	0.000				
**Gangatiri**	0.008	0.000			
**Shahabadi**	0.032	0.031	0.000		
**Purnia**	0.032	0.033	0.033	0.000	
**Siri**	0.027	0.024	0.035	0.044	0.000

**Figure 4:**
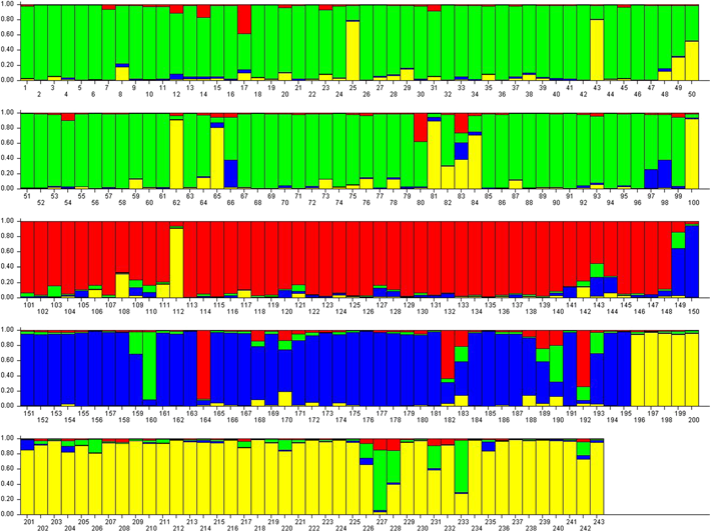
**Clustering assignment of 243 animals representing five East Indian cattle populations using STRUCTURE at K = 4 arranged by Q values.** Each individual cattle is represented on the graph by a vertical bar divided into K colored segments corresponding to K genetic clusters. The length of each colored segment is proportional to the individual’s membership in the cluster of corresponding colour. Shahabadi (Red), Purnea (Blue) and Siri cattle (Yellow) form separate cluster. Bachaur and Gangatiri (Green) cluster in one group.

Among methods not assuming predefined structure, tree-based methods use genetic distance between individuals and tree construction algorithm such as UPGMA or Neighbour-joining to group them in clusters. Similarly, multivariate analyses can help in defining clusters of individuals. However these graphical methods are loosely connected to statistical procedures allowing the identification of homogeneous clusters of individuals. An alternative approach to delineate clusters of individuals on the basis of their genotypes at multiple loci was also performed using a Bayesian approach employed in software STRUCTURE. It works by grouping individuals into clusters (K) such that Hardy-Weinberg equilibrium is maximized within clusters. Likely value of K which best captured the variation present in the data was four based on modal value of K versus ΔK distribution (Figure 4) following Evano et al. (2005). Siri, Purnea and Shahabadi were grouped in their own clusters. However, Bachaur and Gangatiri animals were intermingled in one cluster (Figure 4). The results are coincident with genetic distance among the populations as divergence was lowest between Bachaur and Gangatiri. It is important to mention that geographically habitat of Gangatiri and Shahabadi are much closer than Bachaur and Gangatiri. Closeness of Bachaur and Gangatiri instead of Gangatiri and Shahabadi may be due to attempt in field to upgrade local populations with the use of semen of exotic (Jersey and Holstein Friesian) and indigenous milch (Hariana, Sahiwal, Red Sindhi, Tharparkar) cattle breeds. Secondly the habitat of Gangatiri and Shahabadi are separated by river Ganges, which may act as a geographic barrier. Based on present study Siri cattle appeared to be distinct from all other neighboring breeds and populations of eastern region of India commensurating geographical distance of its habitat resulting in reproductive isolation by distance. As per literature Bhutan is said to be the real home of this breed (Nivsarkar *et al.*^2000^). It was distributed from that area to the various parts of Sikkim and Darjeeling region of West Bengal states. Small cattle with similar black and white markings have been found in Sikong Province of China, which occupies a portion of the Tibetan highlands northeast of Bhutan.

## Conclusions

It is vital to report that lesser known cattle populations too have high genetic diversity. Unfortunately, animals of these populations are continuously decreasing due to change in agricultural practices as mechanization is fast replacing the traditional practices involving these animals, procedures performed by breeders to increase efficiency (crossing with available exotic or crossbred semen) and apathy of government agencies since these populations are not recognized as distinct breeds. The present study established the uniqueness of two such populations; Purnea and Shahabadi from the recognized cattle breeds of Eastern India. Conservation of genetic variation in these populations should be considered by breeders, in the interest of long term future of the populations in their native tract. To begin with, breed societies need to be created, that will be responsible for registration of these cattle populations as breeds, complete maintenance and improvement of the breed to make it economically sustainable in the transforming agricultural scenario of the country.
